# Pravastatin Use and the Five-year Incidence of Cancer in Coronary Heart Disease Patients: from the Prevention of Coronary Sclerosis Study

**DOI:** 10.2188/jea.16.201

**Published:** 2006-09-04

**Authors:** Shinichi Sato, Wakiko Ajiki, Tohru Kobayashi, Nobuhisa Awata

**Affiliations:** 1 Osaka Medical Center for Health Science and Promotion; 2 Osaka Medical Center for Cancer and Cardiovascular Diseases; 3 Statistics and Cancer Control Division, Research Cancer for cancer Prevention and Screening, National Cancer Center

**Keywords:** Hydroxymethylglutaryl-CoA Reductase Inhibitors, Neoplasms, Coronary Arteriosclerosis, Follow-Up Studie

## Abstract

**BACKGROUND:**

Although the short-term safety of statins is well established, their potential carcinogenicity in the long term is still being debated. The aim of this study was to investigate the association between statin-therapy and the incidence of cancer in coronary heart disease patients.

**METHODS:**

The subjects were 263 patients with coronary heart disease who were from Osaka prefecture and who were admitted to the Osaka Medical Center for Cancer and Cardiovascular Diseases between September 28, 1991 and March 31, 1995. The five-year cancer incidence among the subjects was checked using the database of the institution-based cancer registry of the hospital as well as the population-based Osaka Cancer Registry. The Cox’s proportional hazards ratios (HRs) of all cancer incidence and observed/expected (O/E) ratios by cancer site were calculated.

**RESULTS:**

Cancer incidence was observed in 17 patients during the follow-up period. Age (HR=1.16 per one year of age) and continuous smoking during the period (HR=5.82 compared to not smoking during the period) were significantly associated with cancer incidence using multivariable analysis. After being adjusted for sex, age, total serum cholesterol level and smoking habit, the HR of cancer incidence with pravastatin use was 0.78 (95% confidence interval: 0.18-3.46). In the O/E analysis, significantly elevated risks were found for bladder cancer in all the subjects (HR=8.93), as well as in the pravastatin use patients (HR=13.76).

**CONCLUSIONS:**

Pravastatin use for 5 years did not indicate an increase in over all cancer risk.

Coronary heart disease (CHD) has recently become the most common cause of death in the industrialized world.^1^ Elevated serum total cholesterol (TC) levels are widely believed to be a risk factor for CHD, based on studies conducted in Europe, the United States,^2^^-^^4^ and Japan.^5^^-^^7^ It has been shown that 3-hydroxy-3-methylgultaryl coenzyme A (HMG-CoA) reductase inhibitors (so-called statins) reduce serum TC levels and decrease the number of both fatal and non-fatal cardiovascular events^8^^-^^13^ and can cause mortality.^8^^,^^11^

On the other hand, an association between low TC levels and an increased incidence of all types of cancer, including cancers of the stomach, pancreas, liver, and bile duct, has been reported from cohort studies.^14^^-^^19^ Although the short-term safety of statins is well established, their potential carcinogenicity in the long term is still being debated.

Few studies have been conducted on cancer incidence in statin-treated Japanese subjects,^20^^-^^22^ whose common types of cancer are different from those of Caucasians. The Prevention of Coronary Sclerosis (PCS) Study was designed as a single-center study to assess the effect of treatment with the HMG-CoA reductase inhibitor pravastatin on the progression and regression of angio-graphically documented coronary atherosclerosis in patients with CHD.^23^^,^^24^ The aim of the present study was to investigate the association between statin-therapy and cancer incidence in the subjects of the PCS Study.

## METHODS

The PCS Study enrolled 329 eligible patients with CHD aged 70 years or younger who were admitted to the Osaka Medical Center for Cancer and Cardiovascular Diseases between September 28, 1991 and March 31, 1995. Patients with malignant disease were not eligible for the PCS Study. Patients were classified into three groups based on serum TC levels: Group I, TC ≥ 220 mg/dL; Group II, TC 180-219 mg/dL; and Group III, TC <180mg/dL. Patients in Group II were randomized into two subgroups, IIa and IIb, and patients in Groups I and IIa were given pravastatin at a dose of 10mg/day. Clinical events were followed for 5 years by monitoring the patients’ visits to the hospital at least once a year. The details of the study are shown in the references.^23^^,^^24^

Out of 329 cases in the full analysis set at entry, 66 subjects who resided outside Osaka prefecture at entry were excluded, leaving a total of 263 subjects. Cancer incidence in the subjects was checked using the database of the institutional-based cancer registry of the hospital, as well as the population-based Osaka Cancer Registry.^25^

The Cox’s proportional hazards ratios of all cancer incidences were calculated after being adjusted for sex, age, total serum cholesterol level, pravastatin medicated, and smoking habits.

‘Pravastatin medicated’ refers to patients in Groups I and IIa who took pravastatin for ≥ 75 % of the study period, and patients in Groups IIb and III who took pravastatin for >25% of the study period. ‘Non-medicated’ refers to the remaining subjects. Smoking habits were ascertained by interview at entry and in the follow-up period. ‘Quit or start’ refers to a subject who did not continuously smoke during the follow-up period. Person-years at risk were calculated from the date of entry until the date of the diagnosis of cancer, date of death, or closing date, i.e. 5 years after entry or the date of last contact, whichever occurred first. Site-specific sex, 5-year age group, and 5-year calendar period incidence rates for each type of cancer among the Osaka residents were applied to the appropriate person-years at risk to obtain the number of cancers to be expected.^26^ A test of significance for the observed/expected (O/E) ratios was calculated using the exact Poisson probabilities and the 95% confidence interval (CI) of the O/E ratios was based on the formulation derived by Rothman.^26^ This study was approved by the ethics committee of the hospital.

## RESULTS

After 1255.5 person-years of follow-up, we documented 17 incident cases of cancer. Four cases, that is, one-quarter of the cancers were diagnosed in hospitals other than the Osaka Medical Center for Cancer and Cardiovascular Diseases. A past history of cancer before the entry was confirmed in 8 patients. These 8 cases were included in this analysis because the result was almost the same when these cases were excluded.

Table 1 shows the adjusted hazard ratios (HRs) of cancer incidence in the subjects. Age (HR=1.16 per one year of age) and continuous smoking during the period (HR=5.82 compared to non-smoking during the period) were significantly associated with cancer incidence. Increased risks were also observed for men (HR=2.03), Group I (HR=2.44), Group III (HR=1.88 compared to Group II), and for subjects who quit or started smoking during the period (HR=2.02). However, these were not statistically significant. Pravastatin use showed a 22% reduction of cancer risk, which is not significant. The univariable adjusted HR of pravastatin use in smoking patients was 0.46 (95% CI: 0.08-2.50), whereas in the quit or start patients it was 1.57(95% CI: 0.30-8.09) and in the non-smoking patients it was 1.38(95% CI: 0.14-13.26).

**Table 1.** Adjusted hazard ratios of total cancer incidence.

		No. of subjects	Univariable Hazard Ratio	Multivariable Hazard Ratio
Sex	Male	215	1.74 (0.40- 7.60)	2.03 (0.44- 9.47)
	Female	48	1.00 (reference)	1.00 (reference)
Age			1.13 (1.02- 1.25)	1.16 (1.05- 1.28)
Group*	I	150	1.66 (0.53- 5.21)	2.44 (0.62- 9.64)
	IIa and IIb	89	1.00 (reference)	1.00 (reference)
	III	24	1.82 (0.33-9.91)	1.88 (0.30-11.82)
Statin^†^	Medicated	179	1.14 (0.40-3.24)	0.78 (0.18- 3.46)
	None	84	1.00 (reference)	1.00 (reference)
Smoking status^‡^	Continuous	52	2.04 (0.64-6.54)	5.82 (1.56-21.75)
	Quit or start	113	1.64 (0.58-4.61)	2.02 (0.55- 7.39)
	None	98	1.00 (reference)	1.00 (reference)

* : Group I, serum total cholesterol (TC) ≥ 220 mg/dL; Group II, TC 180-219 mg/dL; Group III, TC <180mg/dL. Patients in Group II were randomly assigned to IIa (pravastatin allocation) and IIb (control).

† : Medicated, patients in Groups I and IIa who took pravastatin for ≥ 75% of the study period, and patients in Groups IIb and III who took pravastatin for > 25% of the study period; None, remaining patients.

‡ : Smoking status during the follow-up period

95% confidence intervals in parentheses

The observed number of cancers was not significantly different from the calculating base for the expected number of cancer incidences in Osaka residents (Table 2). The O/E ratio was 1.29 for all subjects of both sexes, ranging from 0.74-1.68 by group. The influence of diagnostic bias seems to be negligible in this analysis because only one case was diagnosed within a year of the entry.

**Table 2.** Observed (O) numbers and O/E ratios of total cancer incidence by sex and group.

Group*	Male			Female			Both sexes		
		
	No.	O	O/E	No.	O	O/E	No.	O	O/E
All subjects	215	15	1.29 (0.72-2.12)	48	2	1.32 (0.15-4.77)	263	17	1.29 (0.75-2.07)
I	113	9	1.53 (0.70-2.91)	37	2	1.72 (0.19-6.21)	150	11	1.56 (0.78-2.80)
IIa	36	2	0.94 (0.11-3.38)	4	0	0 (0.00-38.64)	40	2	0.9 (0.10-3.24)
IIb	45	2	0.78 (0.09-2.83)	4	0	0 (0.00-24.38)	49	2	0.74 (0.08-2.67)
III	21	2	1.84 (0.21-6.64)	3	0	0 (0.00-35.38)	24	2	1.68 (0.19-6.07)

* : Group I, serum total cholesterol (TC) ≥ 220 mg/dL; Group II, TC 180-219 mg/dL; Group III, TC <180mg/dL. Patients in Group II were randomly assigned to IIa (pravastatin allocation) and IIb (control).

95% confidence intervals in parentheses

Table 3 shows the observed numbers and O/E ratios by cancer site. A significantly elevated risk was found for bladder cancer in all the subjects (O/E=8.93), as well as in the pravastatin use patients (O/E=13.76).

**Table 3.** Observed (O) numbers and O/E ratios of cancer incidence by statin medication and site.

Site	Statin medicated*		Non-medicated		All subjects	
		
	O	O/E	O	O/E	O	O/E
All sites	12	1.38 (0.71-2.41)	5	1.12 (0.36-2.60)	17	1.29 (0.75-2.07)
Esophagus	0		1	6.32 (0.08-35.18)	1	1.95 (0.03- 10.85)
Stomach	3	1.69 (0.34-4.95)	1	0.94 (0.01-5.25)	4	1.48 (0.40-3.78)
Liver	1	0.63 (0.01-3.49)	0		1	0.41 (0.01-2.26)
Pancreas	1	3.29 (0.01-18.28)	0		1	2.19 (0.03- 12.19)
Larynx	1	10.77 (0.14-59.92)	0		1	6.85 (0.09- 38.09)
Lung	1	0.77 (0.01-4.28)	1	1.42 (0.02-7.89)	2	1.01 (0.11-3.63)
Ovary	1	28.25 (0.37-157.20)	0		1	24.06 (0.31-133.88)
Prostate	1	4.56 (0.06-25.39)	1	11.03 (0.14-61.38)	2	5.85 (0.66- 21.12)
Kidney	0		1	15.97 (0.21-88.85)	1	5.74 (0.08- 31.95)
Bladder	3	13.76 (2.77-40.21)	0		3	8.93 (1.80- 26.10)

* : Statin medicated, patients in Groups I and IIa who took pravastatin for ≥ 75% of the study period, and patients in Groups IIb and III who took pravastatin for > 25% of the study period; Non-medicated, remaining patients.

95% confidence intervals in parentheses

## DISCUSSION

The hazard ratios of cancer incidence for age, smoking status, sex, and total serum cholesterol levels in our study were similar to the results of past studies.^28^^-^^30^ This may be because this study was carried out with a high follow-up rate and high precision of cancer registration. Pravastatin showed a 22% reduction of cancer risk in our study, but it was not significant. When the sample size in each group was 4427, a 0.050 level two-sided log-rank test for the equality of survival curves will have an 80% ability to detect the difference. We decided that the difference in hazard ratios between the univariate and multivariate analyses of statin use was due to the confounding interference of smoking status.

There have been opposing conclusions about the relationship between statins and cancer in several previous reports from Europe and the US, as statins increased or decreased cancer incidence in various cases. A meta-analyses of five randomized controlled trials (4S, CARE, LIPID, WOSCOPS, AFCAPS/TEXACAPS) performed in 2001 concluded that there was no association between statin use over a 5-year period and the risk of fatal and nonfatal cancer.^31^ In a few randomized controlled trials done afterwards, fluvastatin reduced the risk of cancer incidence in patients who underwent coronary intervention (0.77),^32^ new cancer diagnoses were more frequent with pravastatin use in PROSPER (1.28; 0.97-1.68),^33^ and the 6-year incident cancer rates were similar in the pravastatin treatment group and the usual care group in ALLHAT (1.11; 0.89-1.39).^34^ These few nested case-control studies concluded that the odds ratios decreased in a statistically significant manner, with the odds ratio of the incidence of cancer being 0.72 (95% CI: 0.57-0.92) in Quebec, Canada,^35^ and 0.80 (95% CI: 0.66-0.96) in Holland.^36^ In an analysis using the British General Practice Research Database of 1990-2002, the relative risk of using statins was 1.0 (95% CI: 0.9-1.2). On the other hand the risks of untreated hyperlipidemia in patients increased with colon cancer to 1.8 (95% CI: 1.2-2.8), bladder cancer to 1.9 (95% CI: 1.2-3.1) and prostate cancer to 1.5 (95% CI: 1.1-2.0).^37^ The incidence of any specific type of cancer did not rise in the statin treatment group during the 10-year follow-up of the Scandinavian Simvastatin Survival Study (4S).^38^ The relative risk of cancer death was 0.81 (95% CI: 0.60-1.08). It appears that statins may suppress cancer with long term use of over 5 years.

There are still few studies that review the effect of statins according to the cancer site, and we consider this point worthy of future study. A recent case-control study of colon cancer showed that the relative risk by statins was 0.57 (95% CI: 0.44-0.73) after being adjusted for known risk factors,^39^ but these results have limitations due to a low response rate and a lack of incidences of death. In this study, the data were minimal, but no cases of colon cancer were observed, and the O/E ratio for colon cancer in statin-treated patients was 0.00-2.82.

We also found a significantly elevated risk of bladder cancer. Though the data were minimal, it is conceivable that the results were due to accidents caused by repeated calibration. There exists the likelihood that carcinogenesis may be promoted to the urinary system by repeated angiographies in a patient with coronary heart disease. This is an issue to be examined in the future as well.

According to PATE,^21^ pravastatin treatment is considered to be beneficial for its suppression of other causes of death without cardiovascular events in Japan. In KLIS, the relative risk of cancer mortality was 0.81 (95%CI: 0.47-1.38), but the relative risk of cancer incidence was almost the same in the non-medication group, 1.00 (95%CI: 0.70-1.44).^22^ On the other hand, increased cancer mortality was observed in patients in the relatively low cholesterol group compared with the high cholesterol group in JLIT.^20^

Furthermore, the results of the current study are based on the use of pravastatin, which has specificities such as water-solubility and liver affinity, and therefore the results may not be applicable to other statins. However, the current study maintained a high follow-up rate and matched the data to a high precision cancer registration cohort. This report should contribute to any other meta-analyses that aim to provide useful information after analyzing a large sample size.
